# Extremely Aggressive Mesenteric Extragastrointestinal Stromal Tumor: A Case Report and Literature Review

**DOI:** 10.7759/cureus.23108

**Published:** 2022-03-12

**Authors:** Oluseyi Abidoye, Andrew Johnson

**Affiliations:** 1 Internal Medicine, Northeast Georgia Medical Center Gainsville, Gainesville, USA; 2 Oncology, Longstreet Clinic Cancer Center, Gainesville, USA

**Keywords:** clinical case report, mesentery, extragastrointestinal stromal tumor (egist), gastrointestinal stromal tumor (gist), gist

## Abstract

Gastrointestinal stromal tumors (GISTs) are rare tumors with increasing incidence. GIST is the most common mesenchymal tumor of the gastrointestinal tract involving the elderly population with a slow progression. It originates from the interstitial cells of Cajal. GISTs that develop outside the gastrointestinal tract and have no connections with the intestinal walls or serosal surfaces of the gastrointestinal tubular organs are referred to as extraintestinal gastrointestinal stromal tumors (EGISTs). They have similar morphological and immunohistological characteristics as GISTs. Here, we describe a unique case of an extremely aggressive mesenteric GIST in a 44-year-old African American male.

The patient presented to the hospital with complaints of generalized abdominal pain associated with 50-pound weight loss, decreased appetite, and constipation. He underwent computed tomography (CT) of the abdomen and pelvis which showed a large mass along the central mesentery measuring about 15 × 11 cm with adjacent metastatic nodal disease. He underwent a CT-guided biopsy of his abdominal mass with histopathology findings positive for c-kit (CD117) and discovered on GIST-1 (DOG-1) consistent with GIST. Based on TNM staging, his tumor was graded T4 with N1 given nodal involvement placing him as a stage IV. He was referred to an oncologist and was started on neoadjuvant therapy with imatinib. Mesenteric EGISTs, while rare, are known to have a worse prognosis compared to other EGISTs; hence, prompt action must be taken in aggressively treating these tumors. Factors such as mitotic index and tumor size affect the prognosis of mesenteric GISTs.

## Introduction

Gastrointestinal stromal tumors (GISTs) are rare with an increasing incidence. It is the most common mesenchymal tumor of the gastrointestinal tract [1]. GISTs are predominant in the elderly population and have a slow progression [2]. These tumors arise mostly in the stomach (60%) and small bowel (30%) [1]. GISTs that develop outside the gastrointestinal tract and have no connections with the intestinal walls or serosal surfaces of the gastrointestinal tubular organs are referred to as extraintestinal gastrointestinal stromal tumors (EGISTs). EGISTs account for 10% of all GISTs [3]. Several cases have reported primary sites of EGISTs in the omentum, mesentery, pancreas, reproductive tract, hepatobiliary tract, and retroperitoneum [4]. Here, we describe a unique case of an extremely aggressive mesenteric GIST in a 44-year-old African American male.

## Case presentation

A 44-year-old African American male with a history of hypertension and acid reflux disease presented to our hospital with complaints of generalized abdominal pain for two weeks. He reported a 50-pound weight loss over a period of two months along with decreased appetite, night sweats, generalized malaise, and constipation. He denied any family history of cancer. Social history was significant for tobacco smoking of one pack per day for five years and alcohol use of one to two glasses of red wine every other day.

On admission, his temperature was 98°F, blood pressure was 171/88 mmHg, pulse rate was 93 beats per minute, respiratory rate was 20 breaths per minute, and pulse oximetry was 100% on room air. Physical examination was significant for generalized abdominal tenderness with guarding but no rebound tenderness on palpation. Laboratory results on presentation are listed in Tables 1, 2.

**Table 1:** Complete metabolic panel on admission.

Complete metabolic panel	Values	Reference range
Sodium	134	135–148 mmol/L
Potassium	3.8	3.5–5.2 mmol/L
Bicarbonate	25	21–32 mmol/L
Chloride	102	100–110 mmol/L
Blood urea nitrogen	24.0	3.0–23.0 mg/dL
Creatinine	1.68	0.80–1.30 mg/dL
Glucose	128	65–99 mg/dL
Aspartate aminotransferase	37	0–48 U/L
Alanine aminotransferase	32	13–61 U/L
Total protein	8.2	6.0–8.3 g/dL
Albumin	2.9	3.4–5 g/dL
Total bilirubin	0.50	0.20–1.00 mg/dL
Calcium	8.8	8.4–10.6 mg/dL
Alkaline phosphatase	126	40–150 U/L
Anion gap	7.0	4.3–12.3 mmol/L
Estimated glomerular filtration rate	56.3	>60.0 mL/minute/1.73m^2^

**Table 2:** Complete blood cell count with auto-differential on admission.

Complete blood cell count	Values	Reference range
White blood cell	12.2	4.8–10.8 K/µL
Red blood cell	3.04	4.70–6.10 M/µL
Hemoglobulin	5.9	14.0–18.0 g/dL
Hematocrit	21.1	42.0–52.0%
Mean corpuscular volume	69.4	80.0–94.0 fL
Mean corpuscular hemoglobulin	19.7	27.0–31.0 pg
Mean corpuscular hemoglobulin concentration	28.4	33.0–37.0%
Red cell distribution width standard deviation	42.0	36.4–46.3 fL
Platelets	962	130–400 K/µL
Absolute neutrophil count	8.81	2.00–8.10 10^3^/µL
Absolute lymphocyte count	2.41	0.75–5.50 10^3^/µL
Absolute monocyte count	0.89	0.00–1.20 10^3^/µL
Absolute eosinophil count	0.04	0.00–0.75 10^3^/µL
Absolute basophil count	0.04	0.00–0.40 K/µL

**Figure 1:**
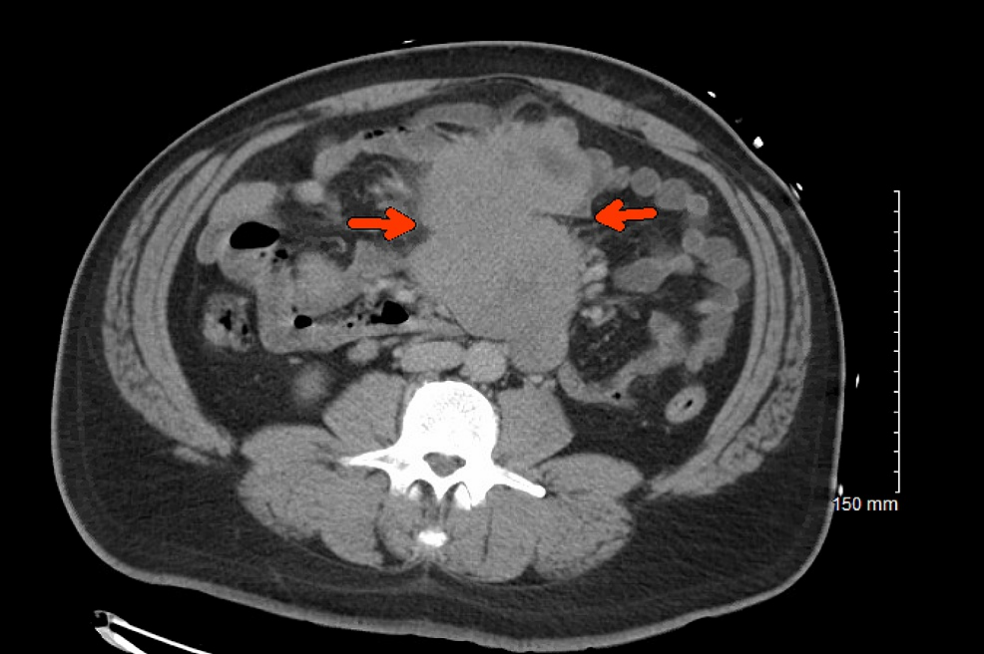
Computed tomography scan showing a large necrotic mass (arrows) along the central mesentery.

**Figure 2:**
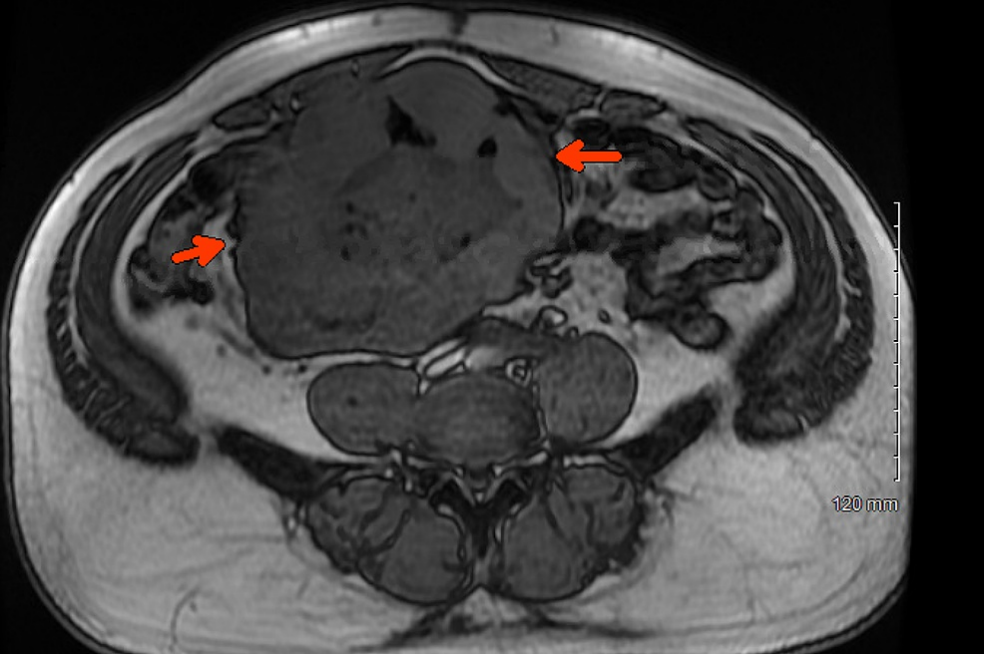
Magnetic resonance imaging of the abdomen showing a large necrotic enhancing mass (arrows) centered in the small bowel mesentery.

Hemoccult blood test was positive. He received two units of packed red blood cells. He underwent computed tomography (CT) of the abdomen and pelvis which showed a large necrotic mass along the central mesentery which closely approximated multiple small bowel loops measuring about 15.5 × 11.9 × 11.6 cm (Figure 1). He underwent magnetic resonance imaging of the abdomen which confirmed adjacent metastatic nodal disease (Figure 2). Tumor markers were also obtained. Carcinoembryonic antigen and cancer antigen 19-9 were within the normal range.

**Figure 3:**
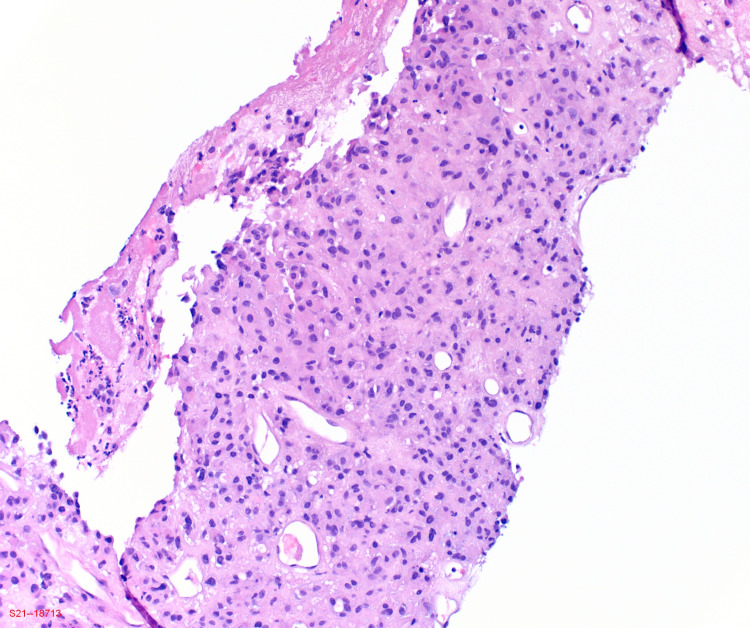
Photomicrograph of low-power histology showing epithelial cells with low-grade nuclear atypia and focal areas of necrosis (hematoxylin and eosin, ×10).

**Figure 4:**
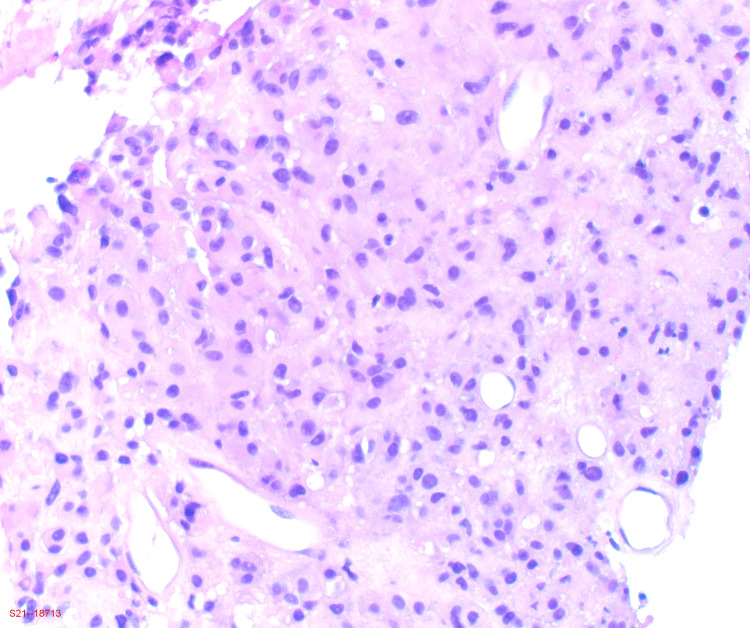
Photomicrograph of low-power histology showing epithelial cells with low-grade nuclear atypia and focal areas of necrosis (hematoxylin and eosin, ×10).

**Figure 5:**
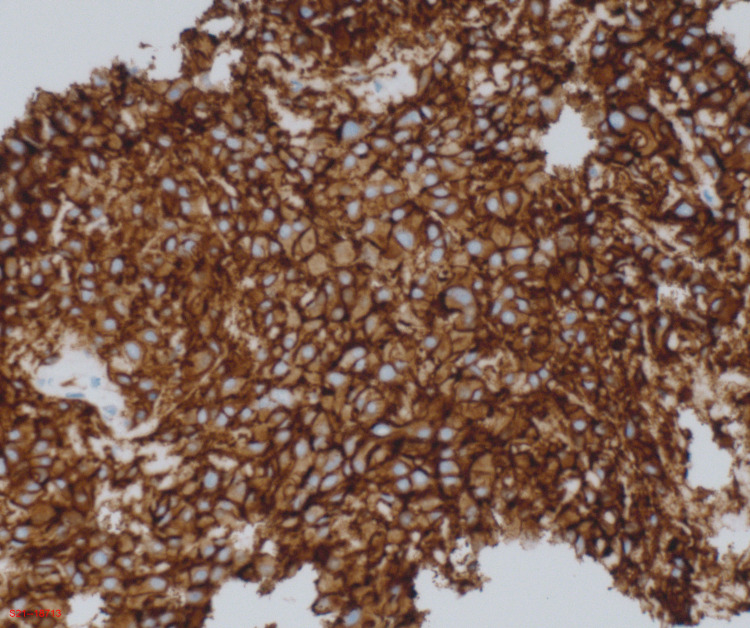
Photomicrograph of immunohistochemical staining positive for CD117 (magnification ×10).

**Figure 6:**
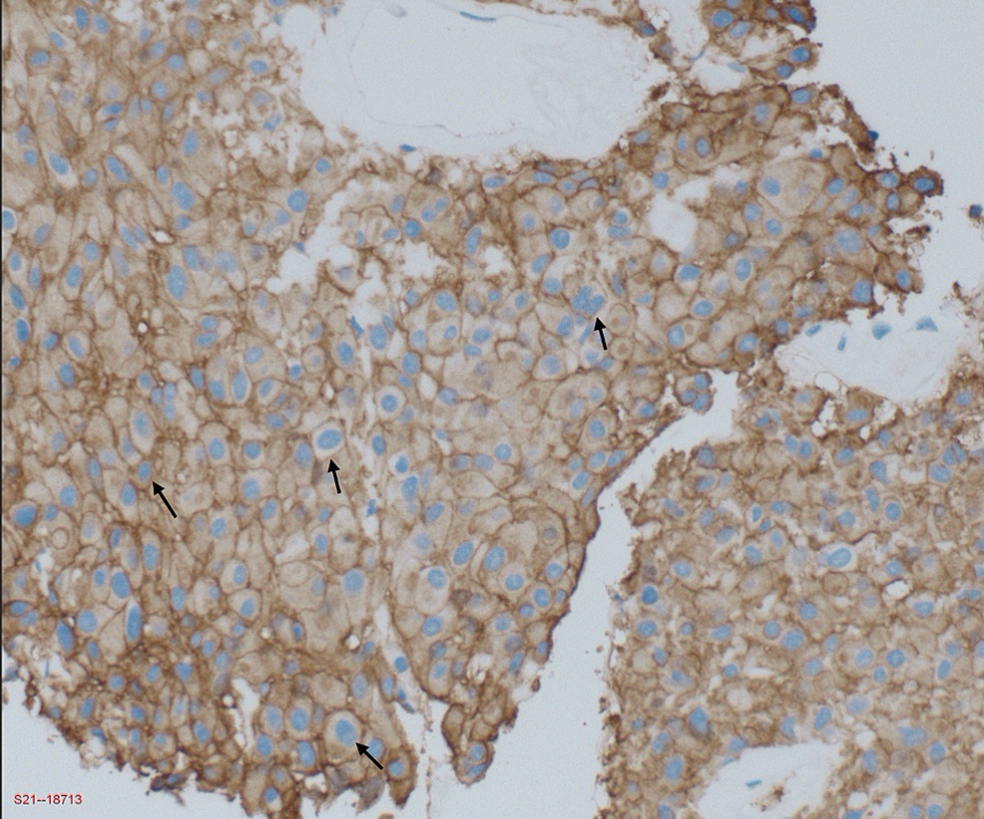
Photomicrograph of immunohistochemical staining was positive for DOG-1 (magnification ×10). Arrows show nuclei with blue staining positive for DOG-1. DOG-1: discovered on GIST-1

Gastroenterology and general surgery were consulted and recommended a tissue biopsy. He underwent a CT-guided biopsy of the abdominal mass. Histological examination revealed epithelial cells with low-grade nuclear atypia with focal areas of necrosis (Figures 3, 4). Mitotic activity was up to three mitoses per 10 high-power fields. Immunohistochemically, the tumor cells showed strong positivity for c-kit (CD117) (Figure 5) and discovered on GIST-1 (DOG-1) (Figure 6). Other immunostains were negative for melanoma, hepatocellular carcinoma, adrenal neoplasm, gastrointestinal, and lung neoplasm. The tumor had expression of Ki-67 proliferation index of approximately 10%. Based on GIST TNM staging, he was graded as stage IV EGIST. Based on the TNM staging, tumor size, mitotic index with a higher risk of disease progression, the patient was referred to a medical oncologist for further management. He was initiated on imatinib 400 mg daily with a good response, which was followed by exploratory laparotomy with resection of the tumor as well as small bowel resection with hand-sewn anastomosis. The procedure was tolerated with no complications. Pathological findings were consistent for GIST. Clinical and imaging results showed no evidence of disease three months after surgery.

## Discussion

EGISTs are GISTs that have no connections with the intestinal walls or serosal surfaces of gastrointestinal tubular organs. These tumors are extremely rare, accounting for 10% of all GISTs [3,4]. EGISTs have been reported to have similar morphological and immunohistochemical similarities as well as genetic make-up such as the expression of c-KIT and *platelet-derived growth factor receptor alpha *(*PDGFRA*) gene mutations with GIST, but differ in the incidence, pathogenesis, and prognosis with GIST based on recent studies [5].

Miettinen et al. [6] were the first to describe EGISTs in 1999 when they reported a series of omental and mesenteric stromal tumors positive for CD117; however, it was Rittel et al. [5] who described EGISTs as tumors with similar morphological and immunohistochemical features as GISTs.

EGISTs similar to GISTs originate from interstitial cells of Cajal (ICC) [7]. ICC are pacemaker cells that control the peristalsis in the gastrointestinal tract [7]. EGISTs mostly occur intra-abdominally and in the retroperitoneum [8]. In a recent study of 13 cases of EGISTs, it was found that 84.6% of EGIST were in the intra-abdominal cavity [8]. Compared to GISTs, studies have reported that EGISTs occur more in females and typically in older patients with a mean age of 54 [8].

Clinically, symptoms vary depending on the location of the EGIST. Patients may be asymptomatic and incidentally diagnosed during routine examination for other medical conditions or may have typical symptoms such as abdominal pain, abdominal mass, or distension [9]. Feng et al. [9] reported that the majority of patients with mesenteric GISTs presented with abdominal symptoms such as abdominal pain, abdominal mass, or distension. In our case, our patient presented with abdominal pain, a common symptom, and anemia which was unusual as EGIST involved the mesentery and not the intestinal walls.

Diagnosis is through tissue acquisition with immunohistochemical staining directed to the expression of the KIT protein, a receptor tyrosine kinase protein seen on ICC [10]. Regarding their genetic make-up, studies have shown similarities with GISTs [5]. Similar to GISTs, most EGISTs express *KIT* gene mutations and in some cases *PDGFR *mutations [5]. Feng et al. [9] examined the clinicopathological features and prognosis of mesenteric GIST in 114 mesenteric GISTs. Among the 18 tumors sequenced, 27.8% harbored a *KIT* mutation and 38.9% a *PGDFRA* mutation, different from typical GISTs in which *KIT *is mutated in 75% cases and *PDGFRA *is mutated in 8-10% of cases [9].

Histologically, EGISTs express markers such as CD34, neuron-specific enolase, smooth muscle actin (SMA), desmin, and S-100 [11]. EGISTs have certain diagnostic markers like GISTs distinguishing them from other sarcomas. These markers include C-kit (CD117), DOG-1, and PKC-0 [5,12]. Reith et al. [5] examined clinicopathological and immunohistochemical features of 48 EGISTs. The tumors expressed CD117 (100%), CD34 (50%), neuron-specific enolase (44%), SMA (26%), desmin (4%), and S-100 protein (4%). In another study involving 114 mesenteric GISTs, out of 50 cases examined, CD117 was expressed in 92%; out of 10 cases examined, 91% expressed DOG-1 [5]. In our case, immunohistochemical report revealed a tumor positive for DOG-1 and CD117.

Treatment for EGISTs depends on staging and risk stratification [9,13,14]. Typically, for locoregional or localized tumors, the standard treatment involves complete surgical excision of the tumor [9]. For locally advanced, inoperable, or metastatic disease, standard treatment is imatinib, a tyrosine kinase inhibitor [9,15]. According to the Armed Forces Institutes of Pathology criteria and the modified National Institutes of Health criteria, the tumor in our patient was classified as highly aggressive recurrence with need for neoadjuvant therapy with imatinib [15,16]. Our patient had locally advanced EGIST and was initially not a candidate for surgical resection. He was placed on tyrosine kinase inhibitor, imatinib, based on guidelines [16], and eventually underwent surgical resection with no evidence of disease after three months. The tumor cells in this case were strongly positive for CD117 and DOG-1.

Mesenteric GISTs have poorer outcomes compared to classical GISTs [10]. In a pooled case series in China, mesenteric GISTs compared to gastric GISTs had poor outcomes with fewer survivals of more than five years compared to gastric GISTs [10]. Several prognostic factors for EGISTs such as size, mitotic activity, and cellularity were identified in the study [10]. Feng et al. [10] noted that tumor size, histological type, and mitotic index were different from GISTs and prognosis of mesenteric GISTs was worse than that of gastric GISTs. The study also noted most mesenteric GISTs exceeding 10 cm in diameter, 5/50 HPF in the mitotic index were high risk. Interestingly, Reith et al. [5] and Yamamoto et al. [17] found that the size of the tumor did not have an impact on clinical outcome, although both studies confirmed that higher proliferation indices were associated with poorer prognosis. Our case was unique based on the age of presentation, the aggressive nature of the tumor, and the treatment approach.

## Conclusions

We presented a case of a rare extremely aggressive mesenteric GIST treated with imatinib and surgical resection. EGISTs are rare tumors that originate outside the gastrointestinal tract and are associated with a worse prognosis compared to GISTs. They can present without any specific symptoms and can grow aggressively resulting in patients presenting late with metastatic disease. Given its rare occurrence and aggressive growth, it is a tumor that should be considered in the list of tumors with clinical relevance that require a multidisciplinary approach, timely recognition, and initiation of prompt treatment to improve outcomes.
